# Magnesium Preserves Calcium Homeostasis and Contributes to Protect Myotubes from Inflammation-Induced Damage

**DOI:** 10.3390/ijms26209912

**Published:** 2025-10-11

**Authors:** Giuseppe Pietropaolo, Sara Castiglioni, Jeanette A. Maier, Federica I. Wolf, Valentina Trapani

**Affiliations:** 1 Dipartimento di Medicina e Chirurgia Traslazionale, Fondazione Policlinico Universitario A. Gemelli IRCCS, Università Cattolica del Sacro Cuore, 00168 Rome, Italy; federica.wolf@unicatt.it (F.I.W.); valentina.trapani@unicamillus.org (V.T.); 2 IRCCS Neuromed, 86077 Pozzilli, Italy; 3 Dipartimento di Scienze Biomediche e Cliniche, Università di Milano, 20157 Milan, Italy; sara.castiglioni@unimi.it (S.C.); jeanette.maier@unimi.it (J.A.M.); 4 Departmental Faculty of Medicine, UniCamillus, Saint Camillus International University of Medical Sciences, 00131 Rome, Italy

**Keywords:** magnesium homeostasis, calcium signaling, skeletal muscle C2C12 cells, inflammation

## Abstract

Magnesium (Mg^2+^) is a key regulator of cellular biochemical processes and an essential cofactor in skeletal muscle physiology. Although Mg^2+^ deficiency has been linked to reduced muscle strength, its role in the regulation of calcium (Ca^2+^) signaling and in inflammation remains incompletely understood. In this study, we examined the effects of Mg^2+^ availability using the murine myoblast cell line C2C12. Cells were differentiated under low, normal, or high Mg^2+^ conditions, and myotube formation, intracellular Ca^2+^ fluxes, and resistance to inflammatory stimuli were assessed. Mg^2+^ deficiency impaired myotube differentiation, while Mg^2+^ supplementation preserved Ca^2+^ response during stimulation and contributed to protect myotubes against inflammation-induced damage. Collectively, these findings highlight a dual role of Mg^2+^ in sustaining functional performance under repeated stress and protecting myotubes against inflammatory injury. This study supports the importance of adequate dietary Mg^2+^ intake as a potential strategy to mitigate muscle loss associated with aging and chronic inflammation.

## 1. Introduction

Mg^2+^, the second intracellular cation by concentration, is one of the most abundant macronutrients in the human body. It plays a crucial role in cellular function, being involved in over 600 enzymatic reactions and numerous metabolic pathways [1]. Approximately 90% of intracellular Mg^2+^ is bound, while free Mg^2+^ concentration ranges from 0.5 to 1.1 mmol/L, which is comparable to serum concentration. The body contains roughly 25 g of Mg^2+^, primarily stored in bone, muscle and other soft tissues [2].

Systemic Mg^2+^ homeostasis depends on concomitant action of intestinal absorption from food and renal regulation of excretion and reabsorption [1]. Mg^2+^ is absorbed through different and concomitant mechanisms: a passive paracellular transport driven by electrochemical gradient and an active transcellular transport mediated by two homologous channels, the transient receptor potential melastatin (TRPM) 6 and 7 [3,4].

Mg^2+^ prevents the dangerous effects of reactive oxygen species (ROS) by inhibiting their production in granulocytes, reducing mast cell degranulation [5], and acting as an anti-inflammatory agent [6]. Studies in animal models demonstrate that low Mg^2+^ levels induce signs of inflammation and an increase in pro-inflammatory cytokines, i.e., IL-1β, TNF-α and IL-6 [5,7]. Several studies have demonstrated that subclinical Mg^2+^ deficiency is associated with elevated levels of C-reactive protein (CRP) and a low-grade inflammatory state, which represents a *primum movens* in the development of various pathological conditions, including cardiovascular disease, metabolic syndrome, osteoporosis, and asthma [8].

Mg^2+^ plays a pivotal role in skeletal muscle physiology and cellular metabolism. Hypomagnesemia has been associated with increased muscle hyperexcitability, hypercontractility, and the occurrence of cramps [9]. During myofiber differentiation, intracellular Mg^2+^ concentration decreases, returning to baseline levels at the end of the differentiation process. This reduction is associated with the downregulation of Mg^2+^ transporters, including magnesium transporter 1 (MagT1) and TRPM7, whose expression remains suppressed even in fully differentiated cells [10]. Throughout this process, Ca^2+^ levels play a critical role: elevated Mg^2+^ concentrations, by altering the intracellular Ca^2+^/Mg^2+^ ratio, promote the formation of larger myotubes compared to those differentiated under low Mg^2+^ [9]. At the cellular level, Mg^2+^ acts as a Ca^2+^ antagonist, sharing the Ca^2+^ binding site on troponin C (TnC) with a significantly lower affinity. Under resting conditions, intracellular Mg^2+^ concentrations exceed those of Ca^2+^, allowing Mg^2+^ to occupy the Ca^2+^/Mg^2+^ binding sites on TnC, thereby stabilizing the relaxed state of the muscle fiber. Following membrane depolarization, Ca^2+^ is rapidly released from the sarcoplasmic reticulum (SR) and replaces Mg^2+^ at TnC binding sites, triggering the contractile response [11]. Moreover, Mg^2+^ is essential for the proper functions of both ryanodine receptors (RyRs), which mediate Ca^2+^ release, and the sarco-endoplasmic reticulum Ca^2+^ ATPase (SERCA), which regulates Ca^2+^ reuptake into the sarcoplasmic reticulum [12].

In vivo and in vitro studies have shown that non-physiological Mg^2+^ levels lead to reduced muscle mass in animal models, accompanied by increased oxidative stress markers and impaired differentiation of muscle stem cells, highlighting the pivotal role of Mg^2+^ in muscle biology [13,14].

In humans, low serum Mg^2+^ levels have been associated with impaired physical performance [15]. A putative protective role for Mg^2+^ in sarcopenia, a common age-associated degenerative muscle condition, has been proposed. Oral Mg^2+^ supplementation has been shown to attenuate muscle damage, potentially reduce intramuscular fat infiltration, and is correlated with lower levels of systemic inflammatory biomarkers [16]. Furthermore, elevated circulating Mg^2+^ concentrations in older individuals have been associated with enhanced muscle function, likely due to the role of Mg^2+^ in mitochondrial energy metabolism and its capacity to counteract ROS-induced damage [17]. Consistently, supplementation with magnesium oxide has also been shown to improve physical performance in elderly populations [18].

Despite the well-established roles of Mg^2+^ in maintaining muscle homeostasis, several aspects remain unclear. For instance, does Mg^2+^ modulate Ca^2+^ signaling and influence myofiber differentiation? Could Mg^2+^ confer protection against muscle injury? In this study, by using an in vitro model, we highlight previously unrecognized functions of Mg^2+^ in preserving muscle responsiveness to depolarizing signals, and in protecting muscle tissue from inflammatory stimuli.

## 2. Results

### 2.1. Mg^2+^ Availability Affects Myotube Differentiation

**Figure 1:**
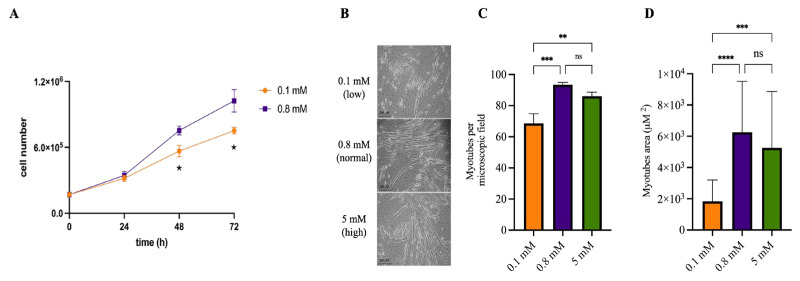
**Mg^2+^ availability affects myotube differentiation.** (**A**) C2C12 cells were seeded in duplicate into 60 mm Petri dishes in media containing low (0.1 mM, yellow) or normal (0.8 mM, purple) Mg^2+^ concentrations. Cells were counted every 24 h. * *p* < 0.05 by unpaired Student’s *t*-test. (**B**–**D**) C2C12 myoblasts were grown to confluence and differentiated in media containing low (0.1 mM, yellow), normal (0.8 mM, purple) or high (5 mM, green) Mg^2+^ concentrations, as detailed in Section 4. Differentiated myotubes were visualized under an optical microscope (10× magnification), and analyzed using ImageJ software. (**B**) Representative images of differentiated myotubes. (**C**) Number of myotubes per microscopic field (mean ± SD) from a representative experiment; three independent fields were analyzed per experiment. (**D**) Myotube area (mean ± SD) from a representative experiment; 10 myotubes were measured in three independent fields. ** *p* < 0.01; *** *p* < 0.001; **** *p* < 0.0001; statistical significance was determined by ANOVA followed by Bonferroni’s post-hoc test. All experiments were performed independently three times.

C2C12 cells can differentiate in vitro, fusing into multinucleated myotubes once confluence is reached and serum is withdrawn. We investigated whether Mg^2+^ availability in the culture medium influences this differentiation process. As expected, reduced Mg^2+^ levels impaired cell proliferation (Figure 1A). Therefore, cells were cultured under normal Mg^2+^ concentrations until reaching confluence, and Mg^2+^ conditions were modified only at the onset of differentiation. As shown in Figure 1B,C, Mg^2+^ deficiency significantly decreased both the number and size of myotubes. In contrast, differentiation in high Mg^2+^ levels resulted in a slightly lower number of myotubes, which did not significantly differ from the control. The Mg^2+^ concentrations used (0.1 and 5 mM) are widely used to model Mg^2+^ deficiency and supplementation, respectively.

### 2.2. Mg^2+^ Availability Affects Ca^2+^ Release from the SR

**Figure 2:**
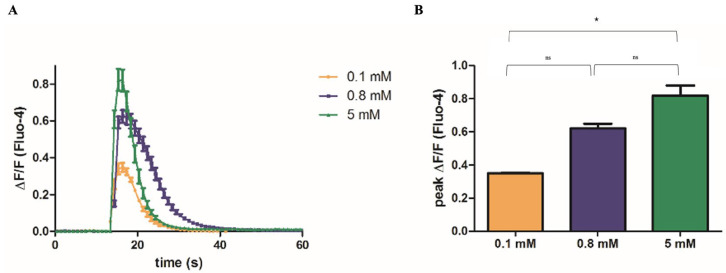
**Mg^2+^ availability affects Ca^2+^ release from the SR.** C2C12 myoblasts were grown to confluence in glass-bottom microscopy dishes and induced to differentiate in media containing low (0.1 mM, yellow), normal (0.8 mM, purple) or high (5 mM, green) Mg^2+^ concentrations, as detailed in Section 4. Intracellular Ca^2+^ concentration was monitored by live confocal imaging in Fluo-4-loaded myotubes. Depolarization was induced at t = 15 s with 50 mM KCl, and single-cell fluorescence changes were quantified by image analysis. (**A**) Time course of the mean relative increase in intracellular Ca^2+^ levels (ΔF/F) ± standard error (SE) from a representative experiment is reported; 10 myotubes were analyzed per experiment. (**B**) The mean maximum amplitude of Ca^2+^ release (peak ΔF/F) ± standard error (SE) is shown for a representative experiment. Each experiment was repeated independently three times. * *p* < 0.05, significance was determined by ANOVA followed by Bonferroni’s post-hoc test.

Excitation–contraction coupling in skeletal muscle fibers relies on rapid communication between membrane depolarization events and Ca^2+^ release from the SR. Experimentally, membrane depolarization can be induced by increasing the extracellular K^+^ concentration. Upon KCl addition, a rapid and transient rise in intracellular Ca^2+^ concentration occurs, followed by a prompt return to baseline levels. The kinetics can be visualized and quantified using live imaging of Fluo-4-loaded cells (see Supplementary Materials, Video S1). We analyzed the kinetics of the Ca^2+^ response in myotubes differentiated under low, normal or high Mg^2+^ conditions. As shown in Figure 2, while the overall kinetics of the Ca^2+^ flux did not differ significantly across conditions, the amplitude of the Ca^2+^ signal positively correlated with Mg^2+^ availability. Specifically, myotubes differentiated in Mg^2+^-deficient medium exhibited a significantly attenuated Ca^2+^ response than those differentiated in Mg^2+^-supplemented conditions.

### 2.3. Mg^2+^ Availability Impairs Intracellular Ca^2+^ Store Refilling

**Figure 3:**
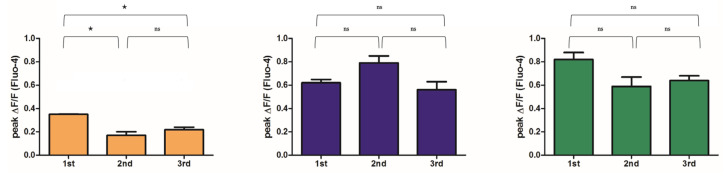
**Mg^2+^ availability impairs intracellular Ca^2+^ store refilling.** C2C12 myoblasts were grown to confluence in glass-bottom microscopy dishes and induced to differentiate in media containing low (0.1 mM, yellow), normal (0.8 mM, purple) or high (5 mM, green) Mg^2+^ concentrations, as detailed in Section 4. Intracellular Ca^2+^ concentration was monitored by live confocal imaging in Fluo-4-loaded myotubes. Depolarization was triggered by 50 mM KCl stimulation applied three consecutive times, each separated by a 10-min recovery period. Single-cell fluorescence changes were quantified by image analysis. The mean maximum amplitude of Ca^2+^ release (peak ΔF/F) ± SE during each stimulation is shown for a representative experiment; 10 myotubes were analyzed per experiment and each experiment was repeated independently three times. * *p* < 0.05, statistical significance was determined by ANOVA followed by Bonferroni’s post-hoc test.

Restoration of baseline intracellular Ca^2+^ levels following excitation–contraction relies on several mechanisms, including active reuptake into the SR. To evaluate the ability of myotubes to sustain repeated cycles of excitation–contraction, we stimulated the same myotubes with KCl, allowed a recovery period of 10 min between stimulations and repeated this protocol three times. As shown in Figure 3, myotubes that had differentiated in control or Mg^2+^-supplemented media maintained a consistent Ca^2+^ response across repeated stimulations. In contrast, myotubes derived from Mg^2+^-deficient cultures exhibited a markedly reduced initial Ca^2+^ response, which declined even further with successive stimulations, indicating impaired Ca^2+^ handling capacity.

### 2.4. Mg^2+^ Availability Affects Myotube Resistance to Inflammatory Stimuli

**Figure 4:**
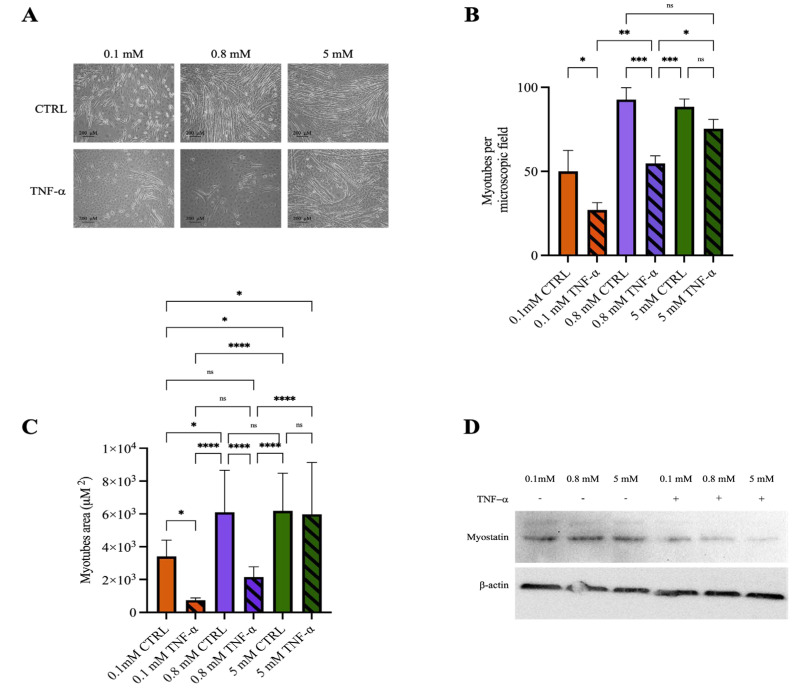
**Mg^2+^ availability affects myotube resistance to inflammatory stimuli**. C2C12 myoblasts were grown to confluence and induced to differentiate in media containing low (0.1 mM), normal (0.8 mM) or high (5 mM) Mg^2+^ concentrations, as detailed in Section 4. After 7 days, differentiated myotubes were either left untreated or exposed to TNF-α (25 ng/mL) for further 24 h. Myotubes obtained under the three Mg^2+^ conditions, with or without TNF-α treatment, were visualized at an optical microscope (10× magnification), and analyzed using ImageJ software on day 8. (**A**) Representative images of control (upper panels) and TNF-α treated (lower panels) myotubes under the indicated Mg^2+^ conditions. (**B**) Number of myotubes per microscopic field (mean ± SD) from a representative experiment (n = 3); three independent fields were analyzed per experiment. (**C**) Mean myotube area (mean ± SD) from a representative experiment (n = 3); for each condition, 10 myotubes were measured in three independent fields. * *p* < 0.05; ** *p* < 0.01; *** *p* < 0.001; **** *p* < 0.0001; statistical significance was determined by ANOVA followed by Bonferroni’s post-hoc test. (**D**) Myostatin expression in myotubes differentiated under the indicated Mg^2+^ conditions, with or without TNF-α treatment, was analyzed by Western blot. β-actin was used as a loading control. A representative blot is shown (n = 2).

Numerous studies have reported the anti-inflammatory properties of Mg^2+^ [5,7,8]. To assess this protective effective of Mg^2+^ in the context of muscular physiology, we conducted preliminary experiments in the C2C12 model and challenged myotubes differentiated under low, normal and high Mg^2+^ conditions with TNF-α, a prototypical pro-inflammatory stimulus (representative images of myotubes prior to treatment are shown in Supplementary Figure S1). As expected, TNF-α treatment markedly disrupted myotubes in both low-Mg^2+^ and control conditions. Notably, myotubes differentiated in high-Mg^2+^ medium appeared largely resistant to TNF-α-induced damage, retaining their morphology and overall integrity (Figure 4A–C). In line with these observations, TNF-α exposure was associated with a more pronounced decrease in myostatin expression—a negative regulator of muscle cell proliferation—in cultures under high Mg^2+^ concentrations (Figure 4D).

## 3. Discussion

Mg^2+^ is an important macronutrient involved in a plethora of pathophysiological processes including oxidative stress regulation, inflammation, and muscle metabolism [1]. Previous findings have indicated that extracellular Mg^2+^ levels influence muscle fiber formation, primarily through modulation of oxidative stress [14]. Consistent with these findings, our results confirm that low Mg^2+^ availability impairs myotube differentiation (Figure 1B), and introduce two novel insights: first, Mg^2+^ availability plays a critical role in preserving functional performance (Figure 2 and Figure 3); and second, Mg^2+^ supplementation confers protection from inflammation-induced myotube disruption (Figure 4). Our findings partially contrast with previous reports suggesting that also Mg^2+^ supplementation can impair C2C12 myotube differentiation [14]. In our experiments, myotubes differentiated in high Mg^2+^ levels were slightly fewer in number, but this difference did not reach statistical significance compared to controls. This discrepancy may stem from variations in the experimental design, including the Mg^2+^ concentrations applied, the composition of differentiation media, and the timing of supplementation. Notably, we observed that myotubes differentiated under high Mg^2+^ levels (5 mM) displayed no differences compared to those cultured under normal concentrations (0.8 mM), indicating that supraphysiological Mg^2+^ concentrations do not further promote differentiation over baseline conditions. In line with our in vitro observations, in vivo studies have shown that subclinical Mg^2+^ deficiency alters muscle fiber characteristics, downregulates Mg^2+^ transporters, and disrupts the expression of genes involved in muscle physiology, metabolism, and regeneration [19].

From a functional perspective, our data indicate for the first time that different Mg^2+^ levels critically influence Ca^2+^ handling in differentiated myotubes. Although the kinetics of Ca^2+^ transients induced by membrane depolarization were comparable across groups, the amplitude of the Ca^2+^ response was markedly reduced under Mg^2+^ deficiency. Notably, during repeated depolarization cycles, Mg^2+^-deficient myotubes exhibited a progressive decline in Ca^2+^ release capacity, suggesting compromised sustainability of excitation–contraction coupling.

This phenotype likely reflects alterations in key components of the excitation–contraction coupling machinery, particularly RyRs, which mediate Ca^2+^ release from the SR to initiate troponin activation and force generation, and SERCAs, which pump Ca^2+^ back into the SR against its concentration gradient [20]. Mg^2+^ is indispensable for the proper function of both RyRs and SERCAs [12,21]. Mg^2+^ serves as a key regulator of RyRs function, acting both as an essential cofactor for ATP-dependent regulation and as a physiological inhibitor of uncontrolled Ca^2^ leak from the SR. Under Mg^2+^ deficiency, RyRs channels may display altered gating behavior, leading to diminished Ca^2+^ release upon depolarization or enhanced basal leak, thereby reducing SR Ca^2+^ content available for subsequent contractions. In parallel, Mg^2+^ is essential for the activity of SERCAs, since ATP binding and hydrolysis are Mg^2+^-dependent. Insufficient Mg^2+^ may therefore impair SERCA-mediated Ca^2+^ reuptake, slowing relaxation and progressively depleting SR Ca^2+^ stores during repeated stimulation. Studies in rats fed an Mg^2+^-deficient diet reported increased RyRs sensitivity to Ca^2+^ and impaired Ca^2+^-ATPase activity in isolated SR, leading to Ca^2+^ overload [22]. The functional impairments we observed may also involve store-operated Ca^2+^ entry (SOCE), a pathway essential for refilling SR Ca^2+^ stores, which is known to decline in aged skeletal muscle contributing to reduced contractile force [23,24]. Mg^2+^ is essential for mitochondrial function, sustaining electron transport chain activity and ATP synthesis [1]. Deficiency leads to mitochondrial damage and increased ROS production [25], which, in turn, can impair Ca^2+^/Mg^2+^ homeostasis and exacerbate RyR and SERCA dysfunction, ultimately reducing Ca^2+^ release capacity during repeated stimulation.

We also demonstrate that Mg^2+^ supplementation protects skeletal muscle cells against inflammation-induced damage (Figure 4), in agreement with its well-documented anti-inflammatory properties [5,26] and with epidemiological evidence linking low dietary Mg^2+^ intake to accelerated skeletal muscle mass loss across multiple cohorts [16,27,28]. In our model, myoblasts differentiated under high Mg^2+^ concentrations were uniquely resistant to TNF-α-induced damage, whereas cells cultured under normal or deficient Mg^2+^ levels were highly susceptible. Mechanistically, the anti-inflammatory role of Mg^2+^ has been attributed to multiple pathways, including the suppression of ROS generation, the inhibition of NF-κB signaling, and the consequent reduction in the expression of pro-inflammatory cytokines such as IL-1β, IL-6, and TNF-α [6,7]. These pathways are central to the cascade leading to muscle damage during inflammation, and Mg^2+^ could act at nodal regulatory points to mitigate cellular injury. Beyond acute protection, Mg^2+^ may also contribute to long-term muscle preservation by maintaining redox balance, stabilizing mitochondrial function, and modulating calcium homeostasis, processes that are critical for sustaining muscle integrity under inflammatory challenges [6,7,29].

Previous studies have shown that the effects of TNF-α on C2C12 cells are dose-dependent. Low to intermediate TNF-α doses markedly impair differentiation by downregulating key myogenic factors, upregulating catabolic genes, promoting atrophy, without causing significant cytotoxicity [30,31,32,33]. At higher concentrations (≥50 ng/mL), TNF-α triggers pronounced myotube atrophy and apoptosis, accompanied by a substantial loss of contractile proteins [34,35,36]. In our preliminary study, designed to evaluate the impact of Mg^2+^ supplementation during inflammation-induced injury, we used an intermediate TNF-α dose to mimic the pauci-inflammatory conditions typically observed in pathological contexts, where Mg^2+^ has been reported to exert protective effects. Notably, we found that, after TNF-α treatment, myostatin expression was reduced in myotubes differentiated under high Mg^2+^ concentrations, compared with those differentiated under low or normal Mg^2+^ levels. These results suggest that elevated Mg^2+^ availability may enhance the resilience of muscle cells to inflammatory stress and potentially support their proliferative capacity. Further studies are required to clarify the molecular mechanisms underlying this protective effect and to determine whether Mg^2+^ primarily counteracts apoptosis, atrophy, or both. Future work should also address the potentially different sensitivity of differentiated myotubes compared with undifferentiated cells to inflammatory stimuli. Finally, an important aspect not examined in this study is the role of Ca^2+^ fluxes under inflammatory conditions. Exploring the interplay between Mg^2+^ and Ca^2+^ homeostasis, using complementary methodological approaches, will be crucial to better understand their cooperative contribution to muscle cell responses [37].

Translationally, our findings suggest that Mg^2+^ deficiency impairs excitation–contraction coupling and Ca^2+^ handling in differentiated muscle cells in vitro, process that may contribute to age-associated alteration in muscle physiology. In older adults or in individuals with chronic or metabolic conditions, Mg^2+^ deficiency may further exacerbate oxidative stress, disturbed Ca^2+^ homeostasis, muscle wasting and frailty. Low dietary Mg^2+^ intake and reduced serum Mg^2+^ levels have contributed to poorer physical performance, decreased muscle strength, and a higher prevalence of sarcopenia in older adults [15,17,38]. Mg^2+^ deficiency is also associated with low-grade chronic inflammation [39,40], which may further accelerate muscle catabolism. Importantly, the improvements in muscle performance observed following Mg^2+^ supplementation in older individuals [18] may be related to mechanisms such as preserved Ca^2+^ handling and protection against inflammation-induced atrophy, as observed in our in vitro model.

In conclusion, while this work was conducted in a single in vitro model and did not include additional mechanistic assays, our results underscore the central role of Mg^2+^ in skeletal muscle biology demonstrating for the first time that adequate Mg^2+^ availability is essential in maintaining a correct Ca^2+^ response to repeated stimulation. Mg^2+^ is critical for myogenesis, the maintenance of calcium homeostasis, and protection against inflammatory stress in C2C12 muscle cells in vitro. While further studies in complementary models and clinical settings are needed to validate and extend our observations, the present results collectively support the idea that adequate Mg^2+^ intake through diet, or supplementation when appropriate, may help maintain muscle health. Based on our in vitro experiments, Mg^2+^ supplementation may represent a practical, safe, low-cost, and effective nutritional strategy to support muscle cell function and resilience to inflammatory stress.

## 4. Materials and Methods

### 4.1. Cell Culture and Differentiation

C2C12 murine skeletal muscle myoblasts were maintained in high-glucose Dulbecco’s Modified Eagle Medium (DMEM; EuroClone, Pero, Italy), supplemented with 20% heat-inactivated fetal bovine serum (FBS), 2 mM L-glutamine, and 100 U/mL penicillin-streptomycin. Cultures were kept at 37 °C in a humidified atmosphere with 5% CO_2_ and used between passages 8 and 12.

To induce differentiation, cells were grown to confluence and then switched to a differentiation medium composed of high-glucose magnesium-free DMEM (Invitrogen, Waltham, MA, USA), 1% FBS, 2 mM L-glutamine, and 1% penicillin-streptomycin. Magnesium sulfate (MgSO_4_) was added to achieve the desired final Mg^2+^ concentrations: 0.1 mM (low), 0.8 mM (normal), or 5 mM (high). Cultures were maintained in differentiation medium for eight days; with medium replaced twice daily to maintain optimal conditions. On day 7, to simulate an inflammatory environment, differentiated cultures were exposed for 24 h to recombinant mouse TNF-α (PeproTech, Cranbury, NJ, USA) at a final concentration of 25 ng/mL. Images of cultured cells were acquired at 10× magnification using an Eclipse TE-2000-S microscope (Nikon Instruments, Campo Bisenzio, FI, Italy). Myotubes were counted in brightfield (phase contrast) images. Image analysis was performed with ImageJ software, version 1.54f. For each experiment, three independent microscopic fields were evaluated. All experiments were performed independently three times, each in duplicate, with similar results.

### 4.2. Western Blot

Cells were lysed in radioimmunoprecipitation assay (RIPA) buffer (50 mM Tris-HCl, pH 8.0, 150 mM NaCl, 1 mM EDTA, 1% NP-40, 0.05% sodium deoxycholate, and 0.1% SDS), supplemented with protease and phosphatase inhibitors (10 µg/mL leupeptin, 20 µg/mL aprotinin, 1 mM phenylmethylsulfonyl fluoride, 1 mM sodium orthovanadate, and 100 mM sodium fluoride). Protein concentration was determined using the Bradford assay (Bio-Rad Laboratories Srl., Segrate (MI), Italy). Equal amounts of protein (50 µg) were separated by 8% SDS-PAGE and transferred onto polyvinylidene difluoride (PVDF) membranes. Membranes were incubated with rabbit polyclonal anti-actin primary antibodies (1:1000, Sigma-Aldrich Srl., Milan, Italy). After stripping with a buffer containing 1 M Tris-HCl (pH 6.8), 10% SDS, and β-mercaptoethanol, membranes were re-probed with rabbit polyclonal anti-myostatin antibodies (1:500, Abcam Ltd., Cambridge, UK). Detection was performed using horseradish peroxidase-conjugated secondary antibodies (GE Healthcare Srl., Milan, Italy) and enhanced chemiluminescence with the ECL Prime Western Blotting Detection Reagent (GE Healthcare Srl., Milan, Italy). Chemiluminescent signals were visualized using the ChemiDoc XRS system (Bio-Rad Laboratories Srl., Segrate (MI), Italy).

### 4.3. Calcium Measurements

C2C12 cells were seeded and differentiated on 35-mm glass-bottom microscopy dishes (Ibidi GmbH, Gräfelfing, Germany), as previously described. For imaging, cells were incubated with 3 µM Fluo-4 AM (Thermo Fisher Scientific, Waltham, MA, USA), a fluorescent calcium indicator, and maintained in a Na^+^, Ca^2+^, and Mg^2+^-free buffer during imaging.

Live-cell imaging was performed using a confocal system Leica TCS SP5 (Tokyo, Japan). The baseline was monitored for 15 s, then KCl 50 mM was added dropwise to induce membrane depolarization. Changes in intracellular calcium levels were quantified by measuring the relative increase in fluorescence intensity (ΔF/F). Image analysis was performed by Leica Confocal Software LCS Lite 2.61.1537, and 10 representative myotubes were examined in each microscopic field. Experiments were repeated independently three times with similar results.

### 4.4. Statistical Analysis

Prism software (version 9.3.1, GraphPad Software Inc., La Jolla, CA, USA) was used for statistical analyses. Statistical significance was evaluated using unpaired Student’s *t*-test or one-way ANOVA, followed by Bonferroni’s test. Differences were considered statistically significant for *p* values < 0.05.

## Data Availability

The row data supporting the conclusion of this article will be made available by the authors on request.

## Supplementary Materials

The following supporting information can be downloaded at: https://www.mdpi.com/article/10.3390/ijms26209912/s1.
