# Supplementary figures and images for: Magnesium Preserves Calcium Homeostasis and Contributes to Protect Myotubes from Inflammation-Induced Damage
Source: Int J Mol Sci. 2025 Oct 11;26(20):9912. doi: 10.3390/ijms26209912 (PMC12564285; doi:10.3390/ijms26209912)

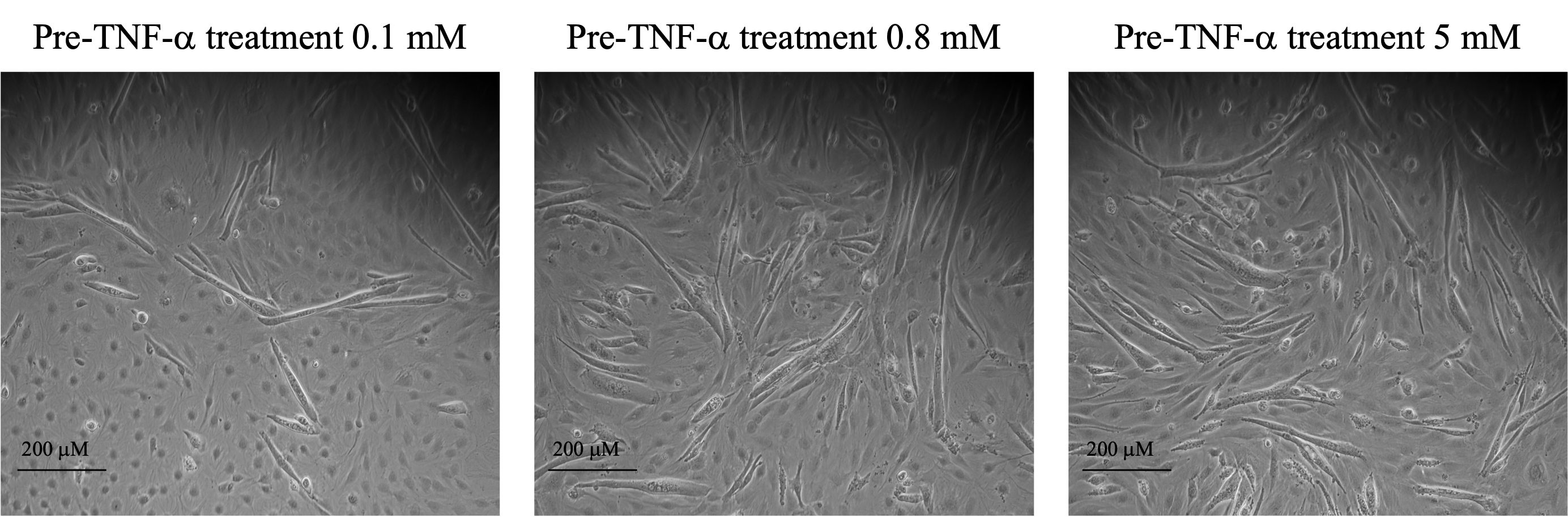

Supplement: Supplementary file 1 [file ijms-26-09912-s001.zip › Figure S1. pre-TNF-α treatment.png]
